# Modeling the Radiative Effect on Microphysics in Cirrus Clouds Against Satellite Observations

**DOI:** 10.1029/2020JD033923

**Published:** 2021-02-19

**Authors:** Xiping Zeng, Jie Gong, Xiaowen Li, Dong L. Wu

**Affiliations:** ^1^ Army Research Laboratory Adelphi MD USA; ^2^ NASA Goddard Space Flight Center Greenbelt MD USA; ^3^ Universities Space Research Association Columbia MD USA; ^4^ Morgan State University Baltimore MD USA

## Abstract

The radiative effect on microphysics (REM) plays an important role in the dew/frost formation near the surface. How REM impacts cirrus clouds is investigated in this study, using bin microphysical model simulations and coincident data of the CloudSat and Global Precipitation Measurement (GPM) satellites. REM affects ice crystal spectrum with two types: radiative cooling and warming. Radiative cooling, as predicted by the bin‐model simulations, favors the formation of horizontally oriented ice crystals (HOICs), but radiative warming does not. Hence, a test of REM can be transformed to a test of HOICs, because HOICs can be measured by the microwave polarization observations of the GPM Microwave Imager (GMI) at 166 GHz. To analyze the GMI data for their HOIC distribution, clouds are sorted into four groups with different optical depth and altitude, based on the radiative cooling/warming ratio (or eta) computed with satellite‐retrieved ice water content. Their HOIC distributions (e.g., the midlevel thick clouds have more HOICs than the high‐level ones) agree well with those predicted by the bin‐model simulations. The general agreement between the GMI observations and bin‐model simulations suggests that REM is common in cirrus clouds and impacts cirrus clouds significantly.

## Key Points

- A microphysical bin model is used to simulate the radiative effect on microphysics (REM) at different altitudes
- The radiative factor of REM is calculated using the satellite‐retrieved ice water content and other variables
- Model results show that REM is stronger in the midlevel thick clouds than in the high‐level ones

## 1 Introduction

Optically thin and thick ice clouds in the upper troposphere affect the Earth's radiation oppositely: the former warms the surface with their inefficient blocking of incoming solar radiation, whereas the latter cools the surface with their strong reflection of solar radiation back to space (Wall & Hartmann, 2018; Zeng et al., 2009). However, these ice clouds are not represented well in the current weather and climate models (Jiang et al., 2012, 2015; Klein et al., 2009; Zhang et al., 2005). For example, the Weather Research and Forecasting model with several sophisticated microphysics parameterization schemes, compared to the W‐band radar observations, tends to generate excessive thick anvil clouds and insufficient thin cirrus clouds (Franklin et al., 2016; Powell et al., 2012; Zeng et al., 2013). The global models have a more serious bias in the same direction (Barahona et al., 2013; Eidhammer et al., 2017; Pincus et al., 2012).

The modeling bias might be mitigated if an additional precipitation process was introduced to convert relatively thick ice clouds to thin ones. One candidate of the precipitation process is the radiative effect on microphysics (REM) (Hall & Pruppacher, 1976; Heymsfield, 1973; Roach, 1976; Stephens, 1983; Wu et al., 2000; Zeng, 2008; see Zeng, 2018a, 2018b, for review).

REM is different from the effect of radiation on clouds through air temperature (i.e., radiation‐modified atmospheric stability and relative humidity). Instead, it is the effect of radiation on cloud particle spectrum via a radiation‐induced difference in temperature between air and cloud particles (see Appendix A). It, for example, can bring about the dew/frost formation near the ground surface even while air is unsaturated (Koeppe & De Long, 1958). It can also bring about diamond dust (or clear‐sky precipitation) in the Arctic regions (Zeng, 2018b) and accelerate the formation of precipitating ice crystals in cirrus clouds via the radiation‐induced broadening of ice crystal spectrum (Gong et al., 2018; Zeng, 2008; Zeng et al., 2019).

REM in clouds is usually noticed by observing its consequence on cloud particles. Stephens (1983), for example, first connected REM to an ice crystal observation: ice crystal can survive a fall of ∼ 6 km through relatively dry air in the upper troposphere (Braham & Spyers‐Duran, 1967). Zeng (2018b) also connected REM to the high frequency of diamond dust observed in the Arctic regions (see Section 4 for more comparison between REM and observations). Can REM be noticed in other clouds via observations? This question falls into the present focus.

Since REM varies greatly in space and time, REM needs to be classified first for a comparison to observations (Zeng, 2018b). REM is classified with the radiative ratio of Zeng (2008, 2018b):

(1) $$\eta_{z}=\frac{F^{+}+F^{-}}{2\sigma T^{4}},$$

where *σ* is the Stefan–Boltzmann constant, *T* is air temperature, and *F* ^+^ and *F* ^−^ are the upward and downward fluxes of infrared radiation (IR) in the atmosphere, respectively. Roughly speaking, *η*_*z*_ is a ratio of total IR warming (numerator) to IR cooling (denominator) of a horizontally oriented plate‐like body (see Appendix A). Since *F* ^+^ and *F* ^−^ are affected by clouds and other factors (e.g., land surface temperature), *η*_*z*_ and thus REM vary from cloud to cloud.

When *η*_*z*_ < 1, ice crystals undergo radiative cooling and thus their spectrum is broadened; otherwise, ice crystals undergo radiative warming and their spectrum is narrowed (see Section 3 for more discussion). Hence, based on the sign of *η*_*z*_ ‐ 1, REM is classified into two types: radiative cooling and warming. In this study, in situ *η*_*z*_ is calculated with satellite‐retrieved cloud variables; once *η*_*z*_ is obtained and then used to classify REM, a microphysical bin model is carried out to simulate REM against observations.

This study consists of six sections. In Section 2, in situ *η*_*z*_ is calculated with satellite‐retrieved cloud variables. In Section 3, bin‐model simulations are carried out to reveal a difference in REM between midlevel and high‐level clouds. In Section 4, the model results are compared with satellite observations. Section 5 discusses other factors of REM (e.g., vertical velocity), and Section 6 concludes with a consistency between the REM modeling and observations.

## 2 Case Study

Since REM varies with cloud height and thickness, a case study is conducted by tracking four clouds (see Table 1). One of them is a midlevel thick cloud. It is compared with other three clouds for the sensitivity of REM to cloud height and thickness. To complement the case study, other clouds are discussed statistically in Section 4.

**Table 1:** *Four Typical Clouds for Analysis*

	Thick cloud	Thin cloud
Midlevel cloud	Cloud A	Cloud B
	(1,322 km, 7 km)	(1,358 km, 7 km)
High‐level cloud	Cloud C	Cloud D
	(170 km, 12 km)	(372 km, 12.5 km)

*Note*. The two numbers in parentheses represent the horizontal distance and altitude of a cloud, respectively, in Figures 1, 2, 3, 4.

### 2.1 Satellite Data Set

The Global Precipitation Measurement (GPM) satellite measures convection and thick clouds via the GPM Microwave Imager (GMI), providing information on the horizontal structure of thick clouds (Hou et al., 2014; Skofronick‐Jackson et al., 2015, 2017). CloudSat measures clouds that range from deep convection to cirrus clouds (Stephens et al., 2002, 2018). The Cloud–Aerosol Lidar and Infrared Pathfinder Satellite Observation (CALIPSO), following the track of CloudSat within the A‐Train, measures thin cirrus clouds such as subvisual cirrus clouds (Winker et al., 2010). Since CloudSat and GPM fly in sun‐synchronous and asynchronous orbits, respectively, they cross each other frequently (Luo et al., 2017; Turk, 2017; Zeng et al., 2019). Their intersections (or coincidences) thus provide plentiful information to evaluate the REM mechanism.

To be specific, CloudSat uses a 94‐GHz (W‐band) nadir‐looking radar to measure clouds, ranging from optically thin clouds to deep convection (Stephens et al., 2002). CALIPSO uses a lidar with 532‐ and 1,064‐nm channels to measure upper‐tropospheric thin clouds (Winker et al., 2010). In contrast, GMI uses 13 passive microwave channels to sense thick clouds and precipitation with wide swaths (∼900 km), 10 of which take both horizontally (H) and vertically (V) polarized measurements at frequencies 10.65, 18.7, 36.5, 89, and 166 GHz. Its high‐frequency channels of 166H and 166V are sensitive to ice crystal scattering, whereas the low‐frequency channels of 36H and 36V are sensitive to liquid drop emission and surface emissivity that is modulated by surface type, wind, temperature, etc. (see Skofronick‐Jackson et al., 2015, for review). The continuous cold brightness temperature observations of 166H and 166V approximately represent the horizontal extension of optically thick ice clouds, whereas those of 36H and 36V represent the horizontal extension of liquid water in deep convection (or mixed‐phase clouds) and warm clouds. Thus, the intersections (or coincidences) of CloudSat and GMI provide information on both vertical and horizontal cloud structure. In the present study, the coincident data set of Turk (2017) is used for cloud analysis, following Zeng et al. (2019).

### 2.2 Case Analysis

**Figure 1:**
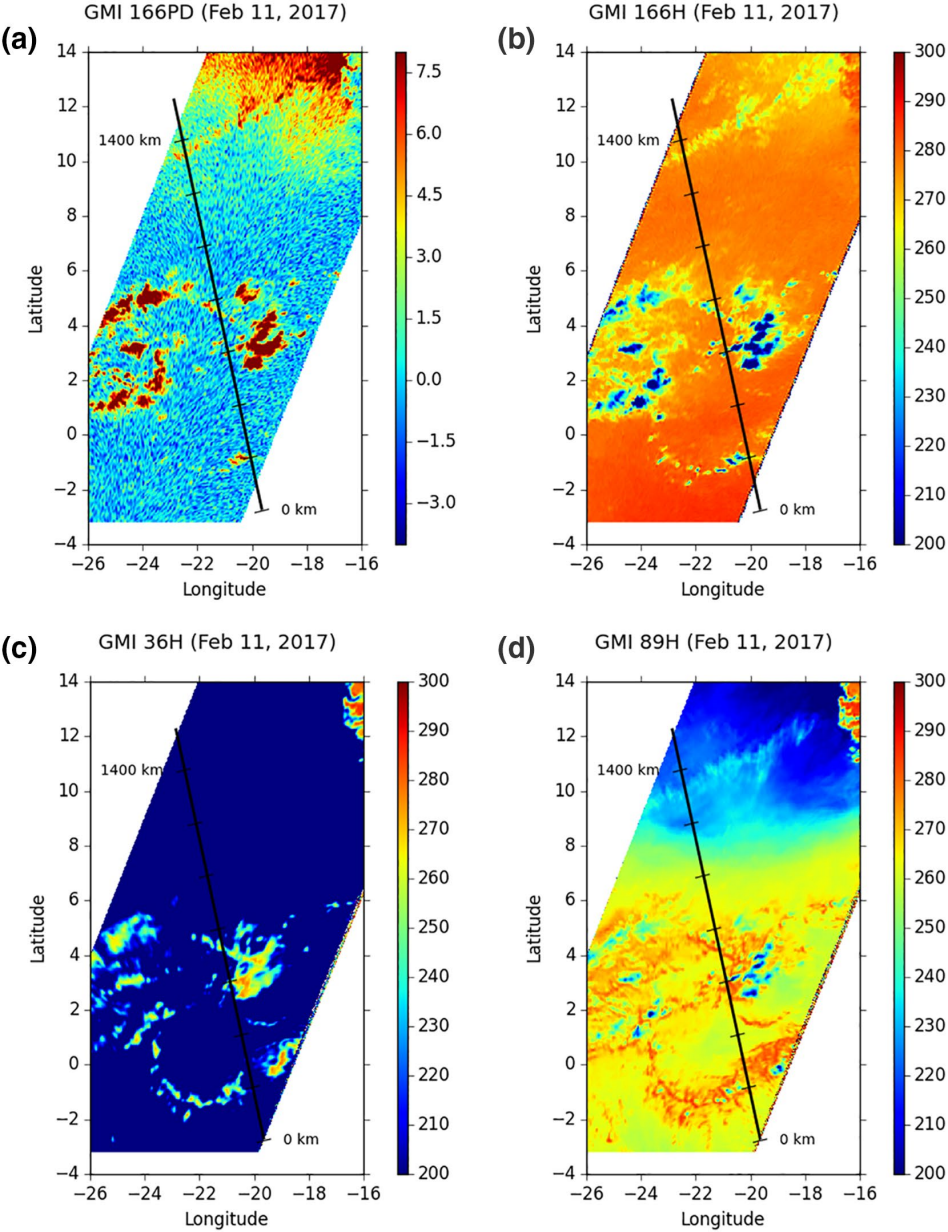
GMI PD (K) at 166 GHz (a), and the brightness temperatures (K) at 166 GHz (b), 36.5 GHz (c), and 89 GHz (d) with the horizontal mode over the Atlantic Ocean at 1506 UTC February 11, 2017. Thick line denotes the CloudSat/CALIPSO track that heads north and is scaled with a unit of 200 km. GMI, Global Precipitation Measurement Microwave Imager; PD, polarization difference; CALIPSO, Cloud–Aerosol Lidar and Infrared Pathfinder Satellite Observation.

One intersection of CloudSat and GMI is chosen for analysis, which occurred over the Atlantic Ocean at 1506 UTC on February 11, 2017. Figure 1 displays the GMI images of brightness temperature (Tb) at 166, 89, and 36.5 GHz with horizontal polarization. It also displays the image of the polarization difference (PD) at 166 GHz, where PD is defined as the difference in Tb between vertically and horizontally polarized channel radiance measurements (i.e., 166PD = 166V − 166H). Since the magnitude of PD at 166 GHz is caused mainly by ice crystal orientations (e.g., Adams et al., 2008; Defer et al., 2014; Roberti & Kummerow, 1999; Skofronick‐Jackson et al., 2008), the 166PD image exhibits the distribution of horizontally oriented ice crystals (HOICs or the ice crystals with long axis oriented in the horizontal plane) near cloud top, given that other ice microphysical properties (e.g., size, habit) are largely homogeneous (Gong et al., 2018; Zeng et al., 2019).

The cloud system near 12°N in Figure 1 is interesting. It brings about high 166PD (Figure 1a), contains no precipitating drops (Figure 1c), and its cloud top is not high (for its warm 166H Tb in Figure 1b). In other words, the cloud system consists of midlevel clouds with an abundance of HOICs.

**Figure 2:**
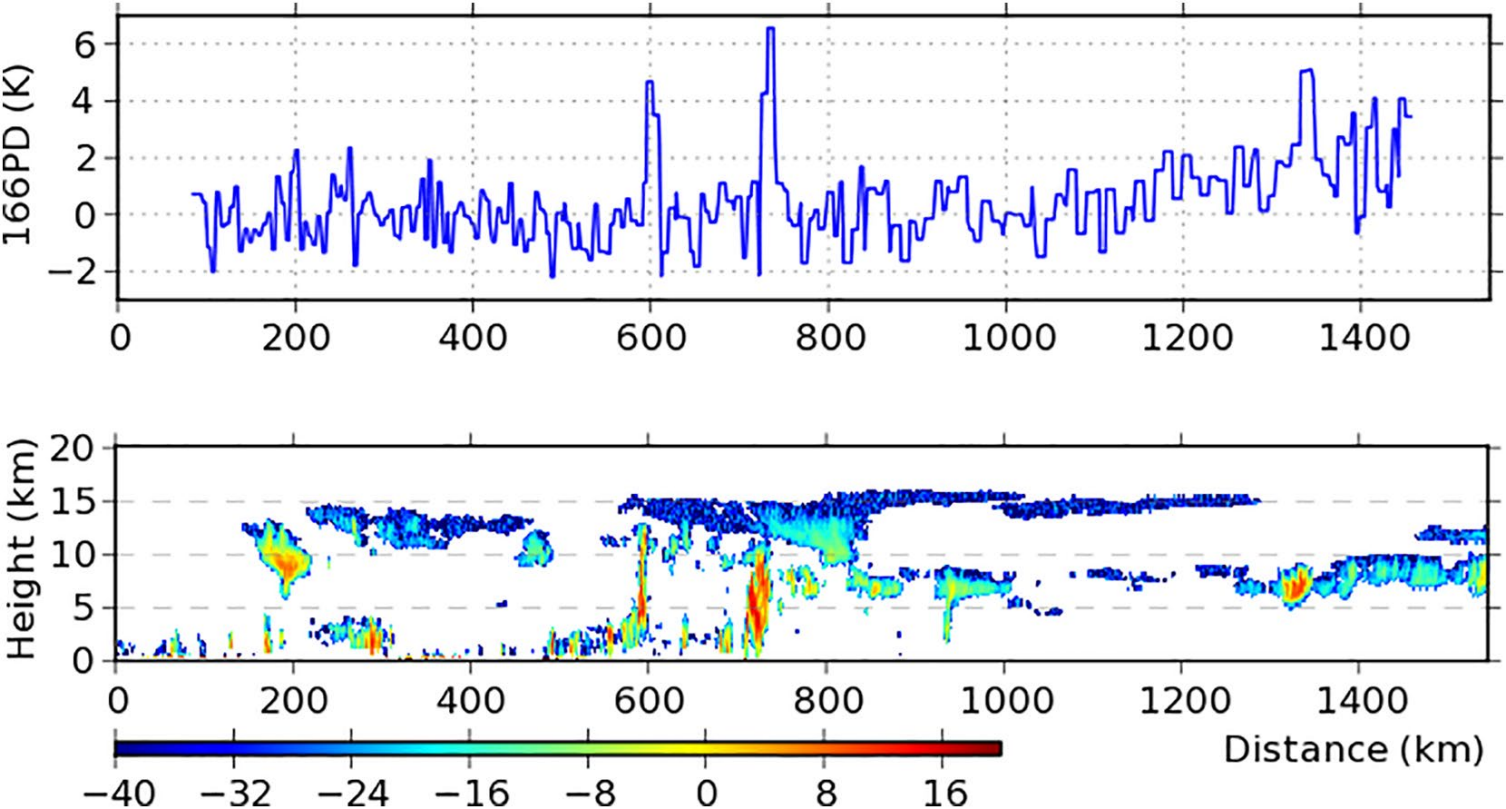
(Bottom) Vertical cross section of the CloudSat radar reflectivity and (Top) GMI 166PD along the CloudSat track shown in Figure 1. Cloud mask for thin clouds is represented by blue, which is obtained from the CloudSat and CALIPSO data (Sassen et al., 2008). Data set starts over 2.69°S and 19.65°W at 15:06:35 and ends over 12.24°N and 22.85°W at 15:10:42 UTC, February 11, 2017. GMI, Global Precipitation Measurement Microwave Imager; CALIPSO, Cloud–Aerosol Lidar and Infrared Pathfinder Satellite Observation.

CloudSat measured the same cloud system. Figure 1 displays its ground track, and Figure 2 displays its vertical cross section of radar reflectivity with the 166PD over the track, showing that the cloud system corresponds to the part between 1,250 and 1,470 km. Figure 2 also shows that the cloud system consists of midlevel clouds with altitude between 5 and 10 km and its clouds correspond to high 166PD, reaching 5 K.

The midlevel clouds in the cloud system contrast the high‐level clouds between 150 and 450 km. The latter occurs between 10 and 15 km altitude with low 166PD (usually less than 2 K). Thus, an interesting question is why the midlevel clouds correspond to high 166PD, whereas the high‐level clouds correspond to low 166PD, which is discussed next with REM.

### 2.3 Computation of In Situ *η*_*z*_

Since the GMI 166PD is sensitive to HOICs near cloud top (or the upper portion of clouds), its high value usually corresponds to an abundance of HOICs near cloud top (Adams et al., 2008; Defer et al., 2014; Roberti & Kummerow, 1999; Skofronick‐Jackson et al., 2008; Zeng et al., 2019). On the other hand, cloud top usually undergoes radiative cooling (or *η*_*z*_ < 1) that in turn brings about HOICs there via REM (Zeng, 2018b; also see Section 4.1). Does the high GMI 166PD correspond to the REM‐induced HOICs? To address the question, it is imperative to compute in situ *η*_*z*_ first.

In situ *η*_*z*_ is computed using Equation 1 and a two‐stream radiation package for *F* ^+^ and *F* ^−^ (Chou et al., 1995; Fu & Liou, 1992). To be specific, *η*_*z*_ is computed with the Goddard radiation package and satellite‐retrieved variables, such as the ice water content (IWC), liquid water content (LWC), and other ancillary atmospheric variables.

**Figure 3:**
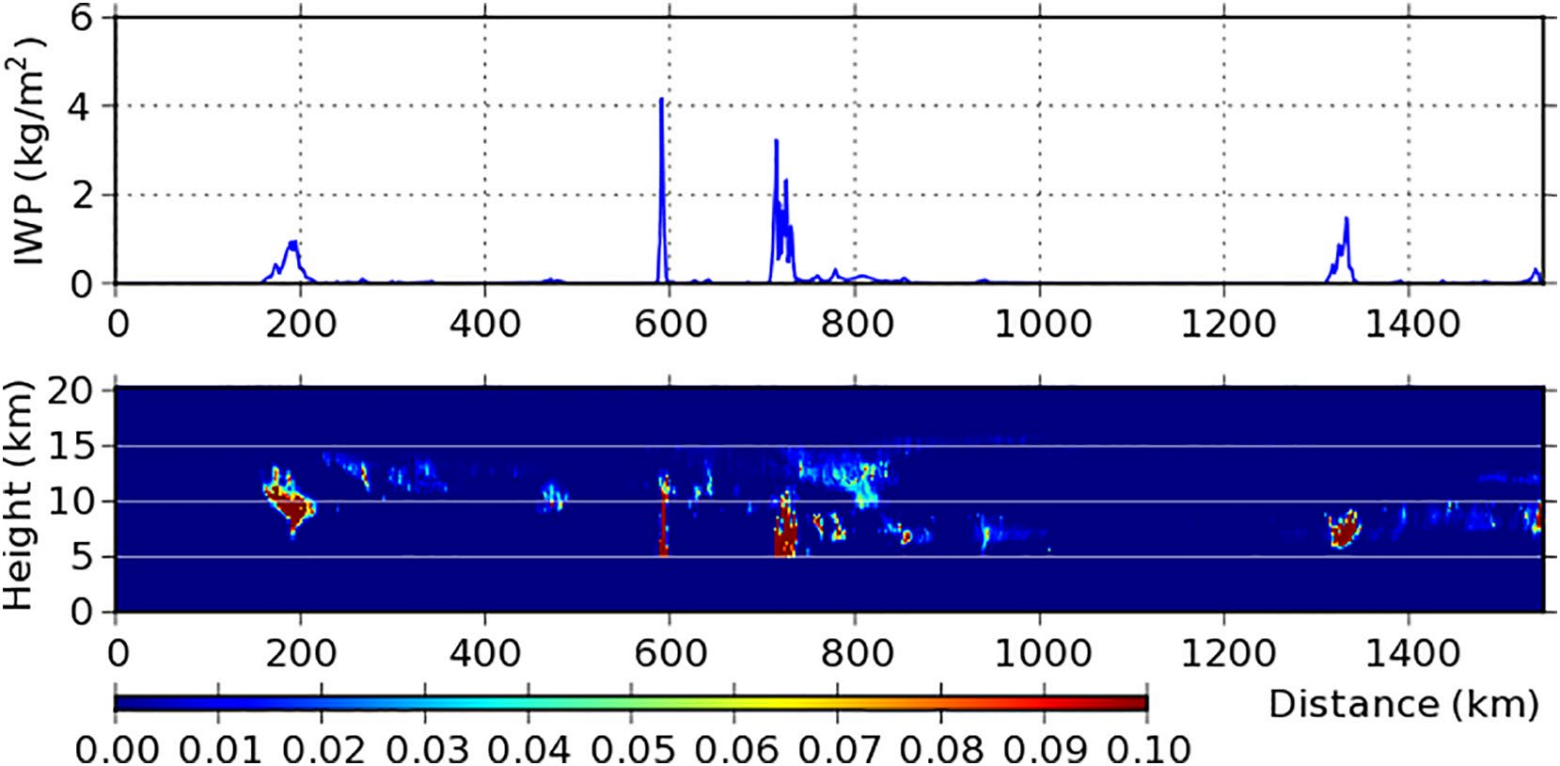
(Bottom) Vertical cross section of retrieved IWC (g/m^3^) and (Top) IWP (kg/m^2^) of the 2C‐ICE product along the CloudSat/CALIPSO track shown in Figure 1. IWC, ice water content; IWP, ice water path; CALIPSO, Cloud–Aerosol Lidar and Infrared Pathfinder Satellite Observation.

The retrieved IWC comes from the 2C‐ICE (CloudSat and CALIPSO Ice Cloud Property) product that combines the data of CloudSat radar and CALISPO lidar (Deng et al., 2013, 2015). Its vertical cross section and the ice water path (IWP) along the track are displayed in Figure 3. In comparison to Figure 2, Figure 3 shows that some thin clouds were missed by CloudSat but caught by CALIPSO, such as those at 12.5 km altitude and 390 km distance.

**Figure 4:**
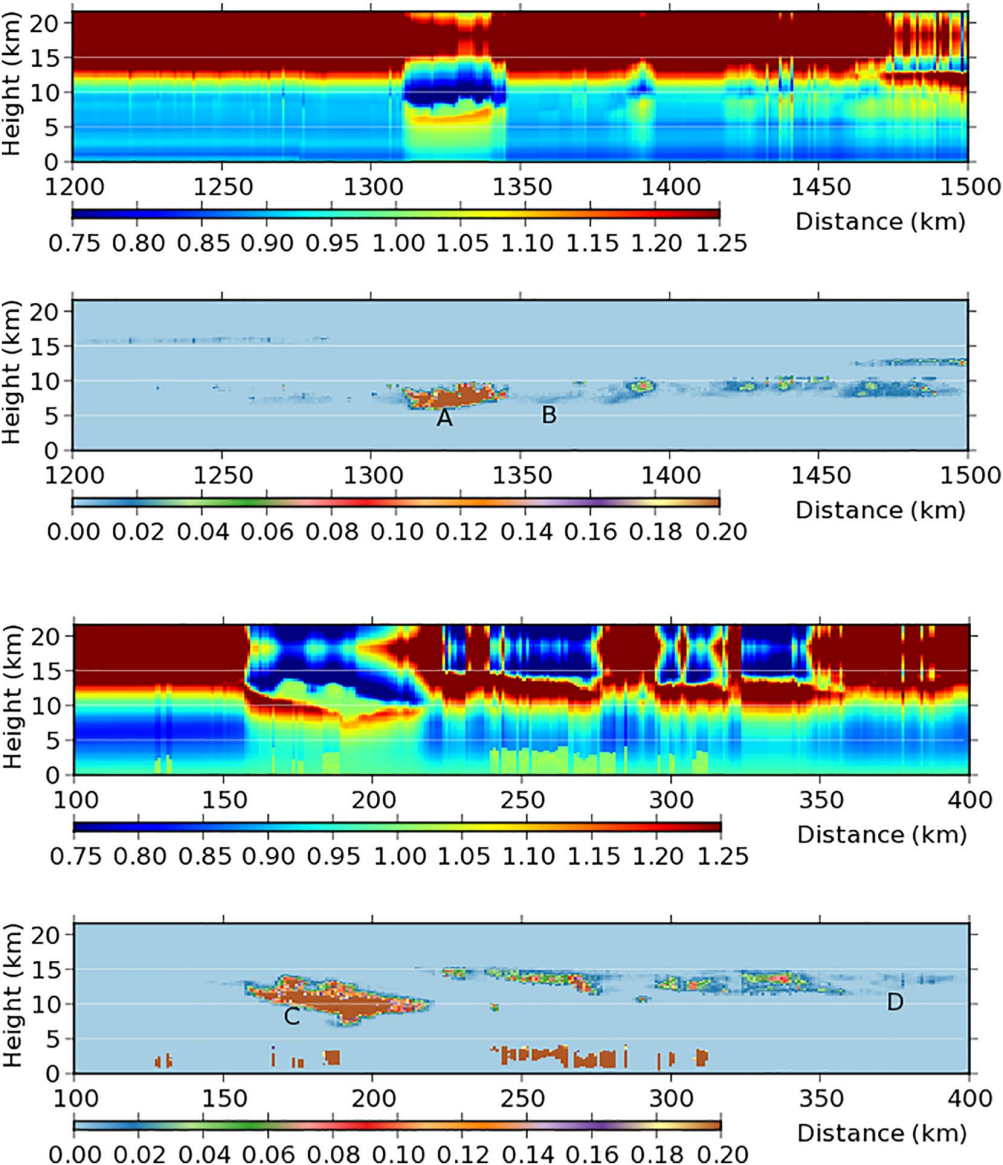
Vertical cross sections of *η*_*z*_ (upper) and the sum of IWC and LWC (g/kg; lower) over two regions: from 1,200 to 1,500 km and from 100 to 400 km. Symbols A–D denote the four clouds analyzed in Table 1. IWC, ice water content; LWC, liquid water content.

The retrieved LWC below the melting layer comes from the CloudSat Radar‐Only Cloud Water Content Product (2B‐CWC‐RO) that is based on the CloudSat radar observations (Austin et al., 2009). In addition, the atmospheric state variables (including air temperature, pressure, specific humidity, and skin temperature) come from the European Center for Medium‐Range Weather Forecasts (ECMWF)‐AUX data set that is based on the ECMWF model. Using these variables as input, in situ *η*_*z*_ is obtained and displayed in Figure 4.

### 2.4 In Situ *η*_*z*_ of Four Typical Clouds

Since *η*_*z*_ varies from cloud to cloud, four typical clouds are analyzed as examples, which are listed in Table 1. The four clouds are marked as A–D in Figure 4.

The thin clouds, B and D, have low IWP (Figure 3). As a result, they have almost the same *η*_*z*_ as their clear‐sky environment (Figure 4), indicating that they change *η*_*z*_ little. In contrast, the thick clouds, A and C, have relatively high IWP (Figure 3). As a result, they alter *η*_*z*_ significantly so that their *η*_*z*_ is quite different from that of their clear‐sky environment. To be specific, *η*_*z*_ < 1 near cloud top and *η*_*z*_ > 1 near cloud bottom (Figure 4). Such a dipole structure of *η*_*z*_ makes sense in physics (Zeng, 2008, 2018a). Near cloud top, the lower part of thick clouds blocks the upwelling IR from the underlying surface, decreasing *F* ^+^ and thus bringing about *η*_*z*_ < 1. Near cloud bottom, ice crystals receive both strong upwelling IR from the underlying surface and strong downward IR from the clouds above, increasing *F* ^−^ and thus bringing about *η*_*z*_ > 1.

In spite of their similar structure of *η*_*z*_, the thick clouds A and C have different 166PD (Figure 2). Cloud A brings about a high 166PD of ∼ 5 K. Such high 166PD over the thick cloud can be explained by *η*_*z*_ < 1 near cloud top (Zeng et al., 2019).

However, thick cloud C as well as its neighboring clouds between 220 and 350 km brings about a relatively low 166PD, although *η*_*z*_ < 1 near its cloud top. Hence, it is inferred that other factors (e.g., air temperature) besides *η*_*z*_ bring about the difference in 166PD between Clouds A and C, which are addressed next using REM modeling.

## 3 REM Modeling

REM depends on two factors: *η*_*z*_ and cloud height. In this section, the microphysical bin model of Zeng (2018b) is used to simulate the second factor or the difference in REM between midlevel and high‐level clouds. Specifically, its numerical experiments are carried out to address why midlevel clouds have more HOICs near cloud top than high‐level ones.

### 3.1 Model Description

The bin model of Zeng (2018b) is used to simulate how REM impacts the ice crystal spectrum in an air parcel. Its input variables include

- vertical velocity *w* that is set to be 0 except for specification,
- *η*_*z*_ that is given to describe the inward radiative flux through parcel boundary,
- air temperature *T* and pressure *p* that are fixed at a given altitude,
- ice crystal shape and orientation that are given but ice crystal size varies via sublimation/deposition, and
- relative humidity RH_i_ with respect to ice that is set to be 100% initially and then changes via ice crystal sublimation/deposition.

In the bin model, *η*_*z*_ is used as an input variable to exhibit REM. However, in a real three‐dimensional weather/climate model, it is computed with upward and downward radiative fluxes and other variables (e.g., air temperature), replicating the interaction between *η*_*z*_ and other processes (including clouds).

Since *η*_*z*_ represents the inward radiative flux through parcel boundary normalized by a scale of energy flux *σT* ^4^ (or the blackbody irradiance at temperature *T*), its magnitude determines the radiative energy budget of each ice crystal in the parcel, although the energy budget of each ice crystal depends on other factors such as ice crystal shape, size, and orientation (see Appendix A for details). In general, *η*_*z*_ is equal to the approximate ratio of the IR flux incident on the surface of a horizontally oriented plate‐like ice crystal to the radiative flux emitted by the same ice crystal.

For an ice crystal in an air parcel with *η*_*z*_, its absorptivity of *F* ^+^ and *F* ^−^ and emissivity as well as *η*_*z*_ are introduced into its energy budget, yielding its diffusional growth rate. Its growth rate is directly proportional to 1 − *η*_*z*_ (Zeng, 2008). When *η*_*z*_ < 1 (radiative cooling), its growth rate is larger than that without radiation; conversely, when *η*_*z*_ > 1 (radiative warming), its growth rate is lower than that without radiation.

For multiple ice crystals in an air parcel with *η*_*z*_ ≠ 1, their spectrum evolution depends on the sign of 1 ‐ *η*_*z*_. When *η*_*z*_ < 1, IR cools all ice crystals in the parcel; as a result, the relatively large ice crystals have a lower temperature than the small ones (see Appendix A for the sensitivity of ice crystal temperature to ice crystal size), altering their competition for water vapor. Their sublimation/deposition adjusts the water vapor pressure of the air parcel *e* so that *e* falls between the saturated vapor pressures of the large and small ice crystals. Subsequently, the large ice crystals grow via vapor deposition, whereas the small ones shrink via sublimate, broadening the ice crystal spectrum (Zeng, 2008; also see Section 3.2). Similarly, when *η*_*z*_ > 1, IR heats all ice crystals in the parcel, and the large ice crystals have higher temperature than the small ones. As a result, the large ice crystals sublimate, providing water vapor to deposit on the small ice crystals, which narrows the ice crystal spectrum (see Section 4.1).

The bin model is used to simulate the REM of multiple ice crystals in an air parcel. Its results thus depend on the sign of 1 − *η*_*z*_. Since *η*_*z*_ varies with altitude (Figure 4), REM varies with altitude as well. On the other hand, given a constant *η*_*z*_, REM varies with altitude via *T* and *p*, too. Suppose that there are two air parcels with *η*_*z*_ = 0.86 and *w* = 0 near the top of clouds A and C, respectively. Their difference in REM thus depends on their difference in altitude (or *T* and *p*), which is studied next using two experiments: H07C and H11C.

### 3.2 REM in MidLevel Clouds

Experiment H07C is designed to simulate REM at altitude *z* = 7 km, where *η*_*z*_ = 0.86, *T* = ‐15.9°C, and *p* = 431 hPa. Since *η*_*z*_ < 1, the experiment simulates the effect of radiative cooling on ice crystal spectrum at *z* = 7 km or near top of the thick cloud A.

The experiment uses 2,048 bins to represent differently sized ice crystals for a given crystal shape and orientation. Its initial relative humidity with respect to ice is 100%. All of the ice crystals are plate‐like and horizontally oriented. Their initial ice crystal spectrum is given by

$$dN\left(a\right)\;=\;c_{1}a\;\text{exp}\left(-c_{2}a\right)da,$$

where *N*(*a*) denotes the number of ice crystals with the semimajor axis length shorter than *a*, and *c*_1_ and *c*_2_ are two constants to be determined with initial IWC and ice crystal concentration (ICC).

**Figure 5:**
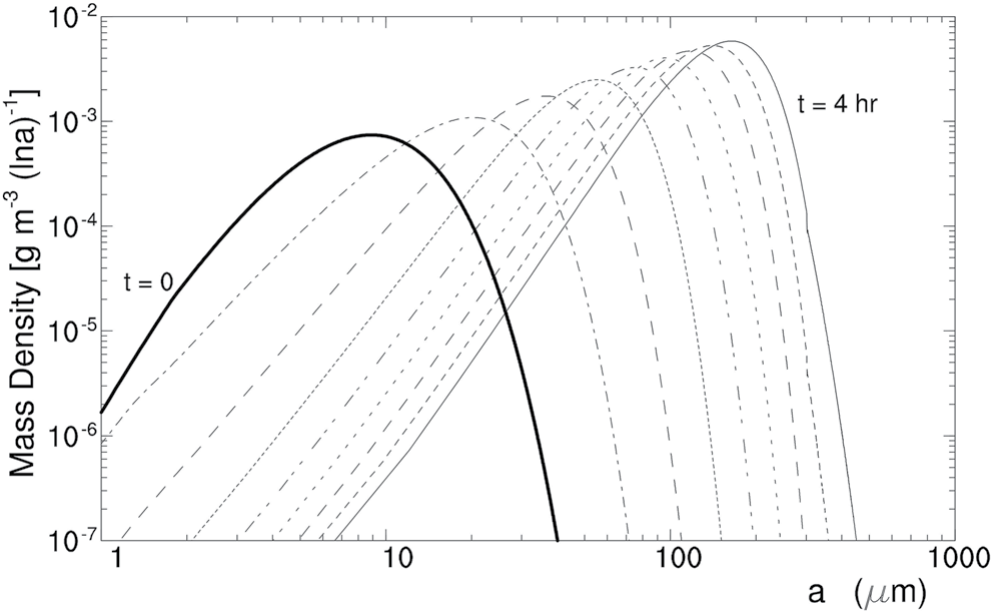
Evolution of mass density d*M*(ln *a*)/d ln *a* versus half‐crystal size *a* in Exp. H07C for HOICs assuming the following parameter values: *η*_*z*_ = 0.86, *T* = ‐15.9°C, and *p* = 431 hPa at *z* = 7 km. Thick line denotes the initial spectrum; time interval between lines is 30 min. HOIC, horizontally oriented ice crystal.

H07C takes the initial ice crystal spectrum with an ICC of 5.0 cm^−3^ and an IWC of 1.5 × 10^−3^ g m^−3^, which is displayed by the thick line in Figure 5. The experiment uses a time step of 0.25 s for model integration and incorporates no solar radiation. All of the ice crystals remain in the parcel without falling out of the parcel even when they are large (see Zeng, 2018b for the effect of crystal fall on ice crystal spectrum).

Figure 5 displays the modeled evolution of the ice crystal spectrum. Its horizontal axis represents half‐crystal size (or semimajor axis length of ice crystal) *a*, and its vertical axis represents d*M*(ln *a*)/d ln *a* where *M*(ln *a*) denotes the mass of ice crystals with half‐crystal size shorter than *a*. The figure clearly shows that radiative cooling broadens the ice crystal spectrum and thus brings about large HOICs.

**Figure 6:**
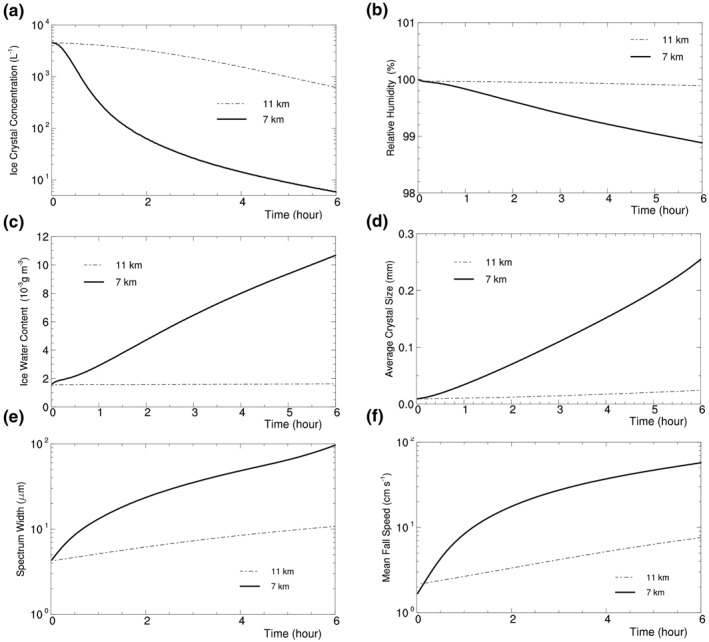
Time series of the number concentration of ice crystals with half‐crystal size larger than 1 μm (a), relative humidity with respect to ice (b), ice water content (c), average ice crystal size (d), ice crystal spectrum width (e), and mean ice crystal fall speed (f) in Exps. H07C (thick) and H11C (thin line). The slight initial difference in mean fall speed comes from the difference in air density between H07C and H11C with the same initial ice crystal spectrum.

Figure 6 displays the number concentration of ice crystals with half‐crystal size larger than 1 μm versus time *t*, showing that the number concentration decreases with time. The figure also displays the time series of IWC, RH_i_, the average ice crystal size

$$\bar{a}=\int am\;dN\left(a\right)/\int m\;dN\left(a\right),$$

ice crystal spectrum width $\sqrt{\int {\left(a-\bar{a}\right)}^{2}m\;dN\left(a\right)/\int m\;dN\left(a\right)}$, and mean ice crystal fall speed

$${\bar{v}}_{f}=\int v_{f}m\;dN\left(a\right)/\int m\;dN\left(a\right),$$

where *m* and *v*_*f*_ are the mass and fall speed of an ice crystal with the semimajor axis length *a*. As shown in Figure 6, IWC increases while RH_i_ decreases with time; in addition, the spectrum width, average ice crystal size, and mean ice crystal fall speed increase with time.

The effect of radiative cooling on the ice crystal spectrum in Figures 5 and 6 can be understood via the relative humidity RH_i_ or the water vapor pressure *e* of the parcel. Since *η*_*z*_ < 1, IR cools all ice crystals in the parcel and decreases the temperature of the relatively large ice crystals more than the temperature of the small ones (Appendix A). As a result, all of the ice crystals possess the saturated vapor pressure below *e*, and thus water vapor deposits on them initially, decreasing RH_i_ and *e*. With the decrease of RH_i_ and *e*, *e* becomes below the saturated vapor pressure of a part of small ice crystals so that the small ice crystals shrink to supply water vapor, while the rest of the ice crystals continue to grow via vapor deposition at expense of water vapor. When the sublimation of the small ice crystals and the vapor deposition on the large ice crystals reach a quasi‐balance in water vapor, RH_i_ and *e* decrease steadily; as a result, the small ice crystals continue to shrink via sublimation while the large crystals continue to grow via vapor deposition, bringing about the broadening of ice crystal spectrum and thus large HOICs.

### 3.3 REM in High‐Level Clouds

Experiment H11C is designed to simulate REM at altitude *z* = 11 km, where *η*_*z*_ = 0.86, *T* = ‐42.9°C, and *p* = 246 hPa. Since *η*_*z*_ < 1, the experiment simulates the effect of radiative cooling on ice crystal spectrum at *z* = 11 km or near top of the thick cloud C.

**Figure 7:**
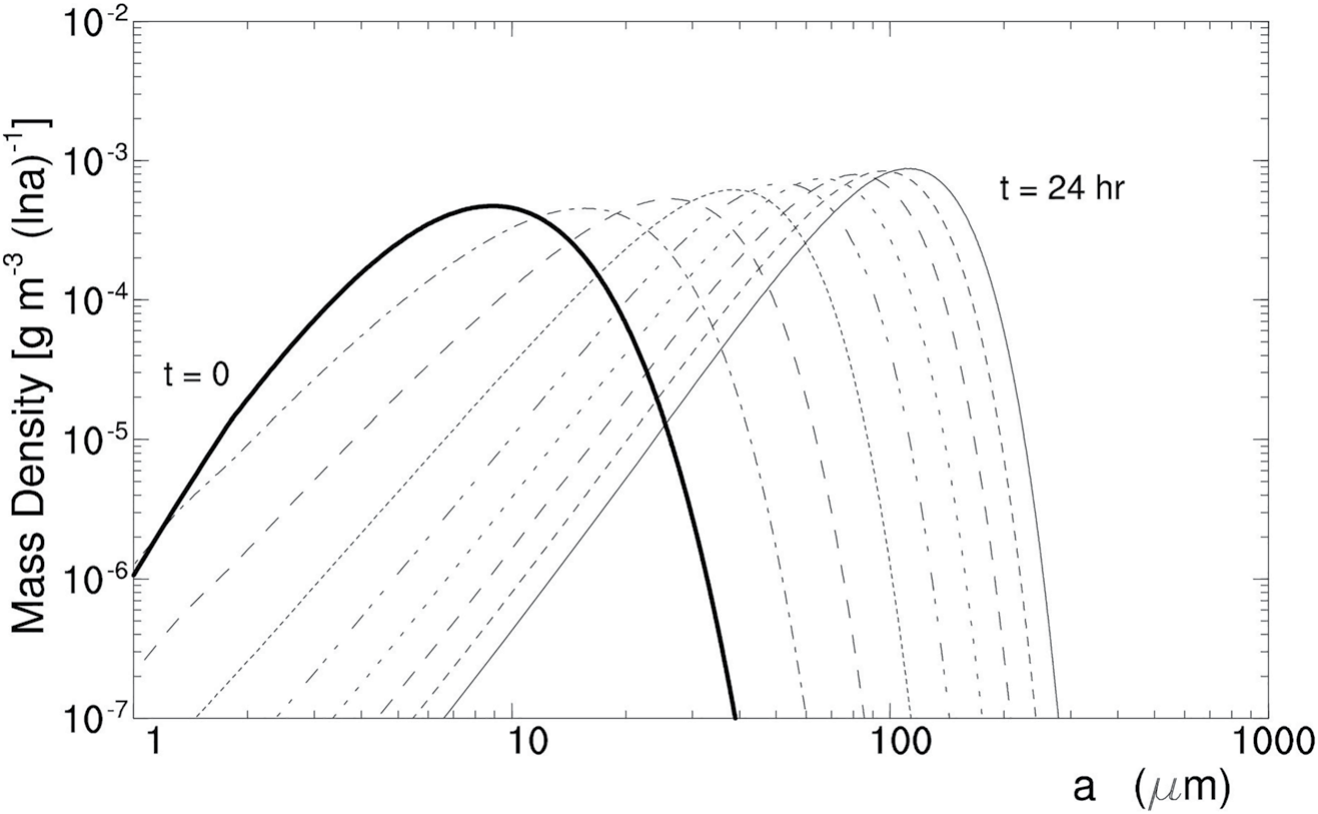
Same as Figure 5 except for H11C at *z* = 11 km and a time interval of 3 h.

The experiment takes the same setup as H07C except for *T* and *p*. It uses a time step of 2 s, which is larger than that in H07C, because its ice crystal growth rate is smaller than that in H07C. Its ice crystal spectrum is displayed in Figure 7, showing again that radiative cooling brings about large HOICs. Its time series of ICC and other variables are displayed in Figure 6. Generally speaking, H11C has a similar spectrum evolution as H07C except that the former has a longer time scale of spectrum broadening than the latter.

H11C and H07C broaden their spectrum with different time scale, because they have different temperature. Their spectrum broadening, as shown by the equation of diffusional ice crystal growth, depends on two factors: supersaturation and the saturation water vapor pressure. Since H11C and H07C have the same *η*_*z*_ and thus almost the same supersaturation, their difference is attributed mainly to the difference in the saturation vapor pressure. Given an increase in temperature, the saturation vapor pressure increases exponentially, bringing about an exponential decrease in the time scale (or the difference between H11C and H07C).

### 3.4 Time Scale of REM

The difference in the time scale between H07C and H11C comes from the effect of *T* and *p* on ice crystal sublimation (Seeley et al., 2019; Zeng, 2008). In this subsection, the time scale at other altitudes is studied with additional experiments (see Table 2 for experiment summary). To be specific, seven other experiments with *η*_*z*_ = 0.86 (i.e., H05C, H06C, H08C, H09C, H10C, H13C, and H15C) are carried out to obtain the time scale of REM at 5, 6, 8, 9, 10, 13, and 15 km altitude, respectively.

**Table 2:** *List of the Numerical Experiments*

Experiment	Altitude (km)	*T* (°C)	*P* (hPa)	*η*_*z*_	Notes
H15C	15	−69.8	131	0.86	Horizontally oriented ice crystals undergo radiative cooling (sensitivity to altitude)
H13C	13	−56.4	181	0.86	
H11C	11	−42.9	246	0.86	
H10C	10	−36.2	285	0.86	
H09C	9	−29.4	328	0.86	
H08C	8	−22.7	377	0.86	
H07C	7	−15.9	431	0.86	
H06C	6	−9.2	491	0.86	
H05C	5	−2.5	559	0.86	
H11C2	11	−42.9	246	0.93	Sensitivity to *η*_*z*_
H07C2	7	−15.9	431	0.93	
HV07C	7	−15.9	431	0.86	Half‐by‐half mixture of horizontal and vertical orientations
HV11W	11	−42.9	246	1.14	
HV07W	7	−15.9	431	1.14	Radiative warming
HV07CU	7	−15.9	431	0.86	Upward motion (*w* = 5 mm/s)
HV07CS	7	−15.9	431	0.86	Ice crystal shape (column like)

To measure the crystal spectrum broadening in bulk, a time scale *τ* is defined as

(2) $$\tau =\frac{t_{100}-t_{0}}{\log_{2}\left({\bar{a}}_{100}/{\bar{a}}_{0}\right)},$$

following Zeng (2008), where the average ice crystal size is increased from ${\bar{a}}_{0}$ at time *t*_0_ to ${\bar{a}}_{100}$ at time *t*_100_. Obviously, the time scale represents a period for ice crystals to double their average size.

**Figure 8:**
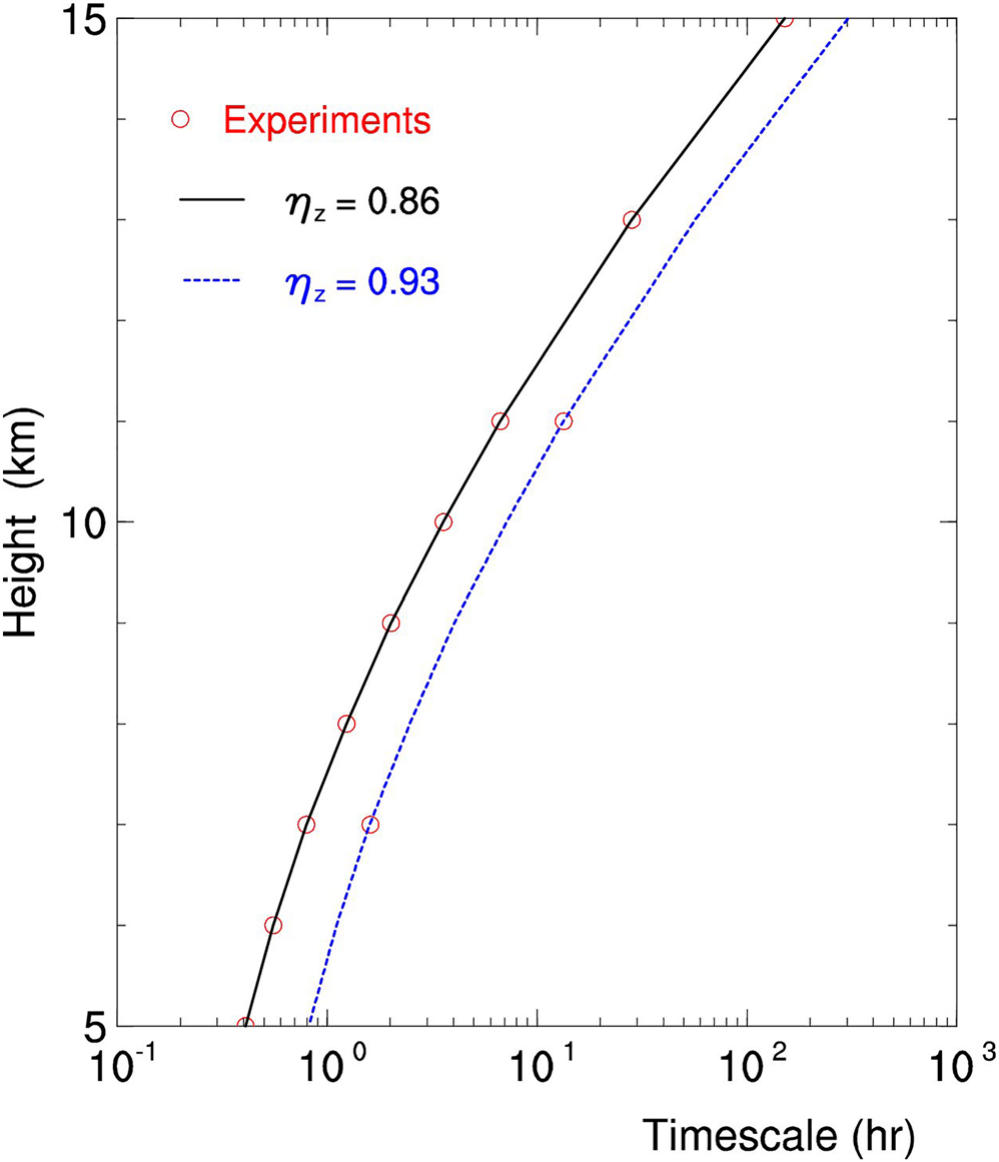
Time scale of REM versus height. A red circle corresponds to a numerical experiment in Table 2. Black line is used to connect the experimental data at *η*_*z*_ = 0.86, and blue line is used to represent the time scale at *η*_*z*_ = 0.93 that is estimated with the experimental data at *η*_*z*_ = 0.86. REM, radiative effect on microphysics.

The time scale *τ* is computed, using the following parameters: *t*_0_ = 0, ${\bar{a}}_{0}$ = 9 μm, ${\bar{a}}_{100}$ = 100 μm, and *t*_100_ that is determined when the modeled average crystal size reaches 100 μm. Using Equation 2 and the results of the nine experiments with *η*_*z*_ = 0.86 in Table 2, the time scale *τ* at nine altitudes is obtained and displayed by the red circles and black line in Figure 8, showing that the time scale increases exponentially with height.

On the other hand, the time scale *τ* depends on *η*_*z*_, which is illustrated with two new experiments: H07C2 and H11C2. H07C2 and H11C2 take the same setup as H07C and H11C, respectively, except for *η*_*z*_ = 0.93. Their modeled time scales are displayed by two red circles in Figure 8, showing that the time scale increases with increasing *η*_*z*_.

In fact, the time scale at the other magnitude of *η*_*z*_ (e.g., *η*_*z*_ = 0.93) can be estimated using the time scale at *η*_*z*_ = 0.86. Since *τ* is inversely proportional to 1 − *η*_*z*_ (Zeng, 2008), the time scale at *η*_*z*_ is approximately equal to the time scale at *η*_*z*_ = 0.86 times (1–0.86)/(1 − *η*_*z*_). The time scale at *η*_*z*_ = 0.93, for example, should be twice the time scale at *η*_*z*_ = 0.86, which is displayed by the blue line in Figure 8. As expected, the time scales obtained from H07C2 and H11C2 are close to the blue line, supporting the estimation of the time scale at other *η*_*z*_ with the time scale at *η*_*z*_ = 0.86.

Based on the connection between *τ* and *η*_*z*_, the spatial distribution of REM near cloud A (or C) can be inferred with the spatial distribution of *η*_*z*_ near the same cloud. On the other hand, although the thick clouds A and C have the similar distribution of *η*_*z*_, they are different in REM (or GMI 166PD) because of their different altitudes (or *T* and *p*) (see Figure 8).

## 4 Modeling Against Observational Statistics

To complement the preceding case study, observational data are analyzed statistically in this section. Since *η*_*z*_ varies greatly with cloud height and thickness, observational data are compared with REM via two steps: sorting observational data based on the sign of 1 − *η*_*z*_ first and then comparing the observational data with their prediction of REM (Zeng, 2018b).

Although the sign of 1 − *η*_*z*_ is determined by multiple factors (e.g., IWC, LWC, air temperature, and land/sea surface temperature), Figure 4 exhibits a “simple” structure of *η*_*z*_: *η*_*z*_ can be decomposed into two parts. That is

(3) $$\eta_{z}={\left(\eta_{z}\right)}_{\text{background}}+{{\left(\eta_{z}\right)}_{\text{cloud}}}_{\;},$$

where ${\left(\eta_{z}\right)}_{\text{background}}$ represents *η*_*z*_ with no cloud and ${\left(\eta_{z}\right)}_{\text{cloud}\;}$ a perturbation of *η*_*z*_ imposed by clouds. In other words, the sign of 1 − *η*_*z*_ is determined by the factors of both ${\left(\eta_{z}\right)}_{\text{background}}$ and ${\left(\eta_{z}\right)}_{\text{cloud}\;}$.

Since ${\left(\eta_{z}\right)}_{\text{cloud}\;}$ represents a perturbation of *η*_*z*_ imposed by clouds, ${\left(\eta_{z}\right)}_{\text{cloud}\;}$is proportional to IWC and LWC approximately. When clouds are optically thin (e.g., clouds B and D), ${\left(\eta_{z}\right)}_{\text{cloud}\;}$ ≈ 0 so that

(4) $$\eta_{z}\approx \;{\left(\eta_{z}\right)}_{\text{background}}.$$

When clouds are thick (e.g., clouds A and C), ${\left(\eta_{z}\right)}_{\text{cloud}\;}$has so large an amplitude that *η*_*z*_ possesses a dipole structure (i.e., *η*_*z*_ < 1 near cloud top and *η*_*z*_ > 1 near cloud bottom).

Based on the decomposition of *η*_*z*_ in Equation 3, clouds are classified into two kinds: thick and thin, using a threshold of infrared cloud optical depth of ∼0.5. Thick clouds possess *η*_*z*_ < 1 near cloud top and *η*_*z*_ > 1 near cloud bottom, no matter where they occur.

Thin clouds may change their sign of 1 ‒ *η*_*z*_ with cloud height and latitude. Cloud B, for example, is at 7 km with 1 ‒ *η*_*z*_ > 0, whereas cloud D is at 12.5 km with 1 ‒ *η*_*z*_ < 0. In view of the opposite sign of 1 − *η*_*z*_ between clouds B and D, thick and thin clouds are divided into midlevel and high‐level clouds, yielding four types of clouds whose samples are denoted as clouds A–D in Table 1, respectively. Next, the observations of the four types of clouds are compared with their prediction of REM, respectively.

### 4.1 Vertical Distribution of HOICs in Thick Clouds

In a thick cloud (e.g., cloud A or C), ${\left(\eta_{z}\right)}_{\text{cloud}\;}$has a dipole structure so that *η*_*z*_ < 1 near cloud top and *η*_*z*_ > 1 near cloud bottom. The dipole structure impacts HOICs at cloud top and bottom oppositely, which is simulated by two experiments of HV07C and HV07W (where C and W denote the radiative cooling and warming of ice crystals), respectively.

**Figure 9:**
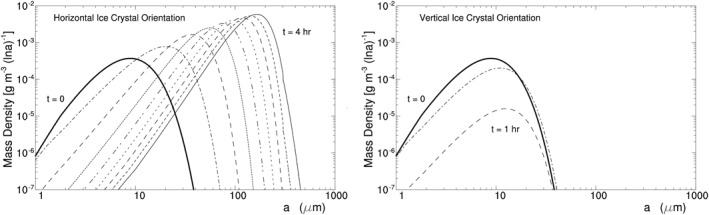
Evolution of mass density d*M*(ln *a*)/d ln *a* versus half‐crystal size *a* in Exp. HV07C for horizontally (left) and vertically (right) oriented plate‐like ice crystals. Thick lines denote the initial spectra; time interval between lines is 30 min.

HV07C takes the same setup as H07C except that half of its initial ice crystals are horizontally oriented and the other half are vertically oriented; this is because turbulence can bring about the random orientation of small ice crystals (Klett, 1995). Its modeled ice crystal spectrum is displayed in Figure 9. HOICs, like those in H07C, grow to form precipitating particles in a few hours. In contrast, the vertically oriented ice crystals shrink due to sublimation and disappear eventually. This preference for HOICs is attributed to radiative cooling, because HOICs lose energy to space more efficiently and consequently have lower temperature than vertically oriented ice crystals at *η*_*z*_ < 1 (see Appendix A for ice crystal temperature vs. orientation).

HV07C suggests that large HOICs form near cloud top due to radiative cooling. Once the large HOICs form, they fall through the cloud while a part of them becomes randomly oriented due to turbulence (Klett, 1995). They eventually arrive at cloud bottom and then undergo radiative warming (or *η*_*z*_ > 1) there. Their fate near cloud bottom is simulated by another experiment of HV07W.

**Figure 10:**
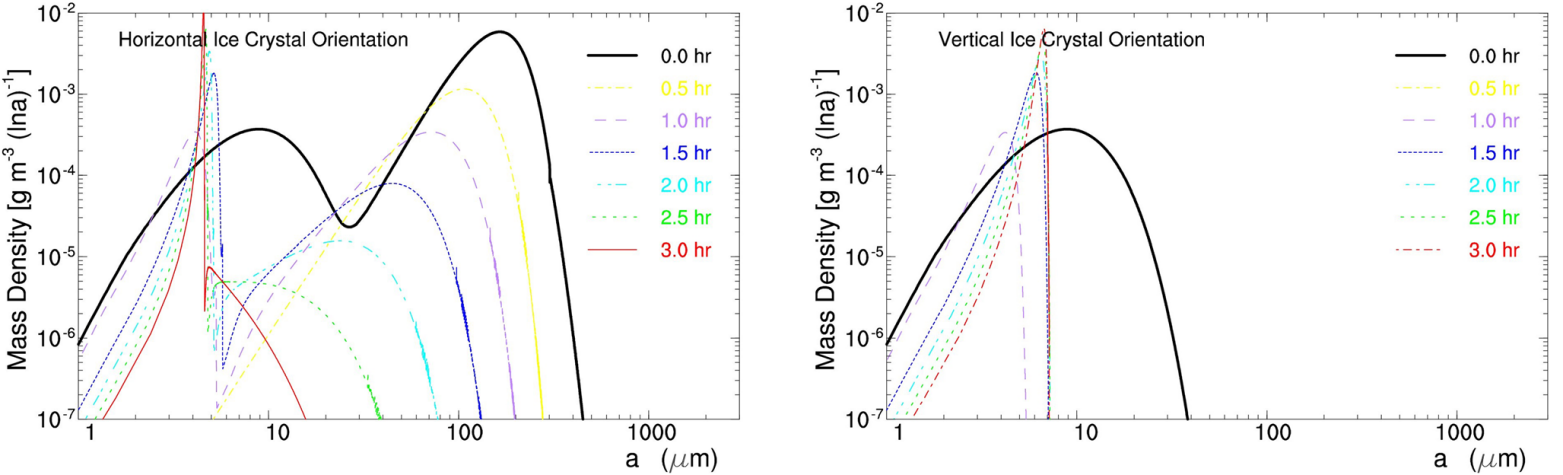
Same as Figure 9 except for Exp. HV07W with *η*_*z*_ = 1.14.

HV07W takes the same setup as HV07C except for *η*_*z*_ = 1.14. Its initial ice crystals consist of two parts: the original small ice crystals in the air parcel and the large ones that fall in from cloud top. To be specific, its initial ice crystal spectrum is equal to that of HV07C plus the spectrum of HV07C at 4 h, which is shown by the thick lines in Figure 10.

The evolution of ice crystal spectrum in HV07W is displayed in Figure 10. The large HOICs shrink via sublimation, because the radiative warming (or *η*_*z*_ = 1.14) increases their temperature more than others. Meanwhile, the small HOICs grow via vapor deposition, because the radiative warming increases their temperature less than others. In addition, the small vertically oriented ice crystals shrink first and then grow. As a result, REM decreases the preference of an ice crystal orientation near cloud bottom, approaching to no preference.

In summary, HV07C and HV07W predict that there are more HOICs near cloud top than near cloud bottom. This prediction on the vertical distribution of HOICs is supported by the lidar observations in the Arctic (Goerke et al., 2017; Neely et al., 2013). The Cloud, Aerosol Polarization and Backscatter Lidar was developed to measure diattenuation with a beam zenith angle of 32°; its strong diattenuation observation near cloud top shows that there are more HOICs near cloud top than near cloud bottom (Goerke et al., 2017; Neely et al., 2013). Such a vertical distribution of HOICs is consistent with the prediction of REM in thick clouds.

### 4.2 Statistical Observations of High GMI 166PD Over Thick Clouds

HV07C and the dipole structure of ${\left(\eta_{z}\right)}_{\text{cloud}\;}$ suggest that there are HOICs near top of thick clouds. Since the GMI 166PD is sensitive to HOICs near cloud top or the upper portion of clouds (Adams et al., 2008; Defer et al., 2014; Roberti & Kummerow, 1999; Skofronick‐Jackson et al., 2008; Zeng et al., 2019), a positive correlation is expected between GMI 166PD and thick clouds.

The positive correlation between 166PD and thick clouds is confirmed by the statistical analysis of GMI data. Zeng et al. (2019) statistically analyzed the 166PD over thick clouds. They found that the 166PD over thick clouds is positive from the tropics to high latitudes, supporting the prediction of REM in thick clouds.

### 4.3 Statistical Difference in GMI 166PD Between Midlevel and High‐Level Thick Clouds

As shown in the case study, the midlevel thick clouds (e.g., cloud A) bring about a high 166PD of 5 K, whereas the high‐level clouds (e.g., cloud C) bring about a relatively low 166PD. In other words, the higher the cloud top, the lower the 166PD. Such a difference in 166PD between the midlevel and high‐level thick clouds is caused by their difference in the REM time scale (or Figure 8).

**Figure 11:**
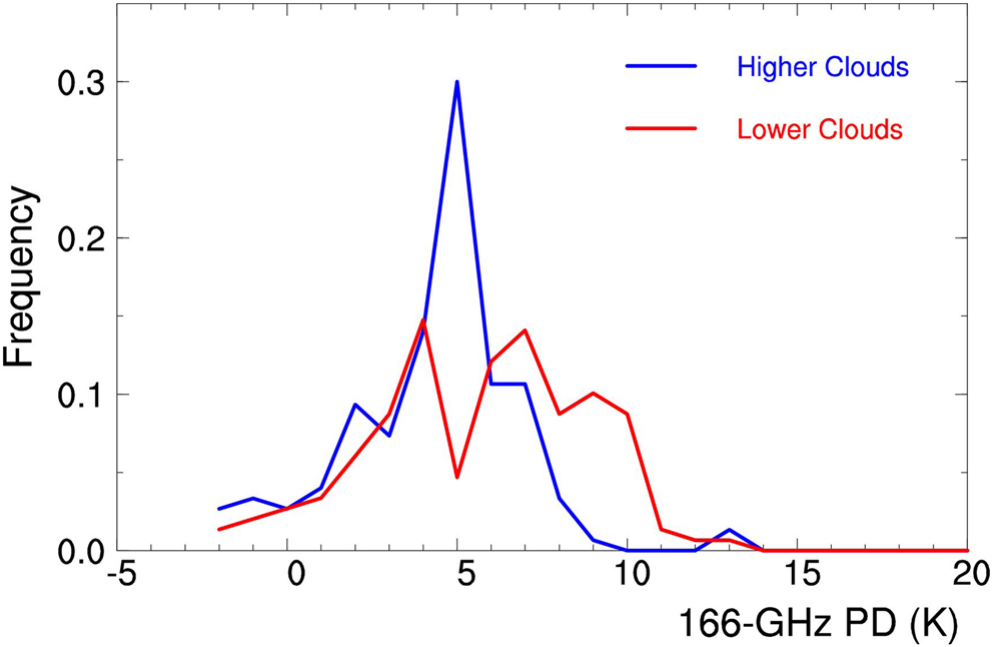
PDF of GMI 166PD (K) over the higher (blue) and lower clouds (red) in the tropics, where the higher and lower clouds represent the clouds with top height above 12 km and below 10.5 km, respectively. PDF, probability density function; GMI, Global Precipitation Measurement Microwave Imager.

This case study is supported by the statistical result of GMI data. Figure 11 displays the probability density functions (PDFs) of GMI 166PD over the higher and lower thick clouds in the tropics, where the PDFs are obtained with 3 years of CloudSat‐GMI coincident data (March 2014 to March 2017) between 20°S and 20°N. Specifically, clouds are chosen for analysis with the following criteria: ice cloud geometric thickness <3 km, IWP in a range of 0.05 and 4 kg/m^2^ (Gong & Wu, 2017), and 166V Tb (or Tb at 166 GHz with the vertical mode) in a range of 160 and 250 K (Gong & Wu, 2017). Such criteria are used to avoid contamination from convective clouds and the cold surface signals with very dry air above (e.g., dark lake or oceans in Arabian–North Africa area).

In Figure 11, the higher and lower clouds represent the clouds with top height above 12 km and below 10.5 km, respectively. As their difference in PDF reveals, the lower clouds have larger 166PD than the higher clouds, supporting the case study.

The PDF distributions in Figure 11 are consistent with other statistical analyses of GMI 166PD (Gong & Wu, 2017; Gong et al., 2018, 2020). Gong et al. (2018) statistically analyzed the GMI 166PD versus 166V Tb in the tropics. When 166V Tb is ∼195 K, 166PD reaches the largest magnitude of ∼8.5 K. With 166V Tb decreasing from 195 to 100 K, 166PD decreases from 8.5 to 1.5 K. If 166V Tb is treated as a variable to approximately represent cloud top level, the correlation between 166PD and 166V Tb implies that the 166PD decreases with increasing cloud top level, supporting the PDF distributions in Figure 11 and the sensitivity of REM to altitude in Figure 8.

### 4.4 CALIPSO Observations

**Figure 12:**
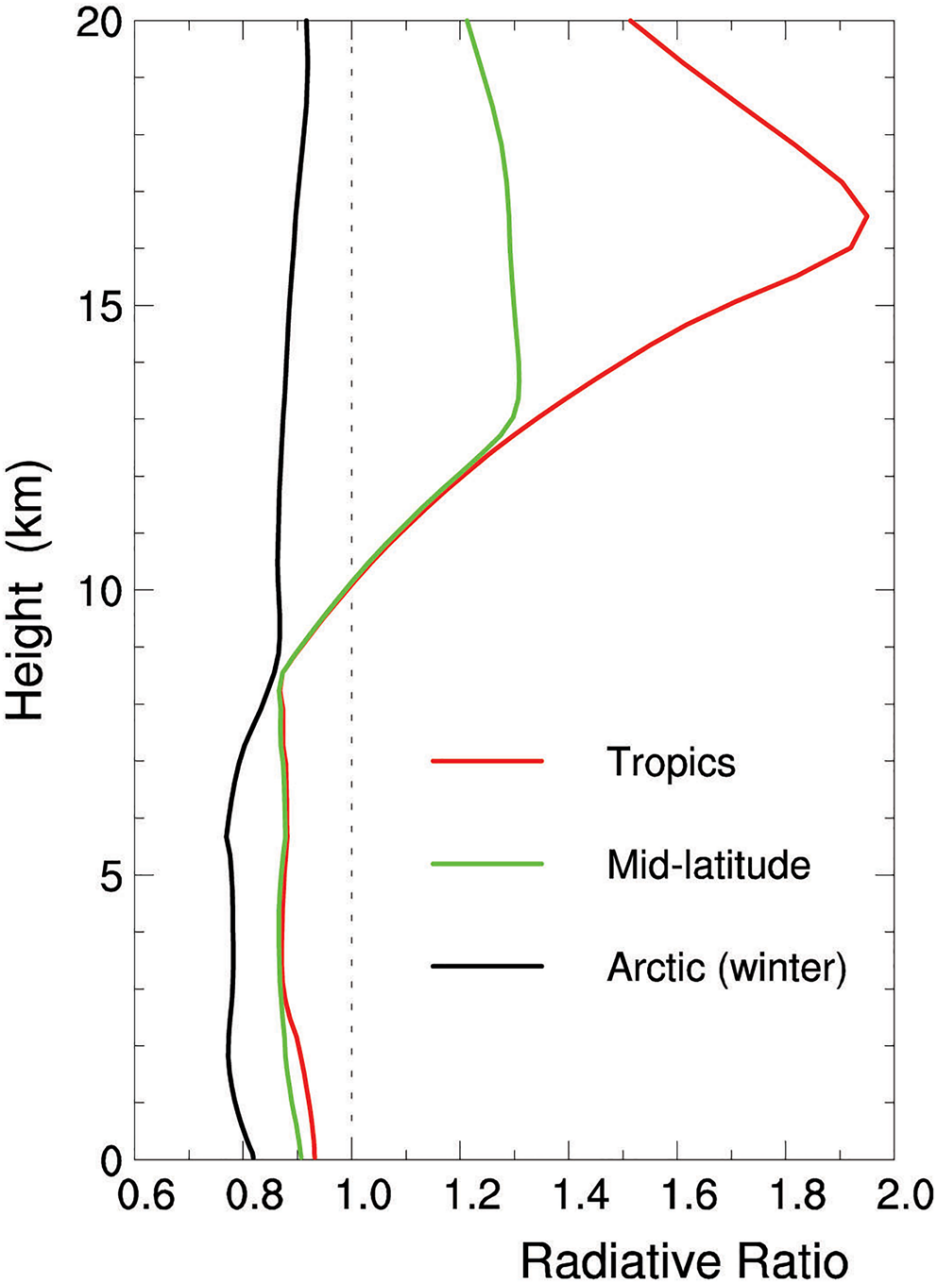
Vertical profile of the radiative ratio *η*_*z*_ with no cloud, ${\left(\eta_{z}\right)}_{\text{background}}$, in the tropics (red), middle latitudes (green), and Arctic regions during wintertime (black).

Thin clouds (e.g., clouds B and D; including the thinnest clouds such as diamond dust) change *η*_*z*_ little so that $\eta_{z}\approx \;{\left(\eta_{z}\right)}_{\text{background}}$. Their value of *η*_*z*_, or ${\left(\eta_{z}\right)}_{\text{background}}$, is computed using Equation 1 and the Goddard radiation package with the data of mean atmospheric states in the tropics, middle latitudes, and high latitudes during wintertime (Chou et al., 1995). Its vertical profiles in the low, middle, and high latitudes are displayed in Figure 12. Generally speaking, *η*_*z*_ < 1 below 10 km and *η*_*z*_ > 1 above 10 km in the tropics and middle latitudes, whereas *η*_*z*_ < 1 throughout the troposphere in the high latitudes during wintertime.

Based on the vertical distribution of ${\left(\eta_{z}\right)}_{\text{background}}$, HV07C predicts that the ice crystals with horizontal orientation have more chance to grow than those with other orientation below 10 km. Another similar experiment, HV11W, is carried out to show no preference for horizontal ice crystal orientation above 10 km.

HV11W takes the same setup as HV07C except for *η*_*z*_ = 1.14, *T* = ‐42.9°C, and *p* = 246 hPa. Thus, one‐half of its initial ice crystals are horizontally oriented and the other half are vertically oriented. It simulates how radiative warming impacts the thin clouds at 11 km (e.g., cloud D). Its results show no obvious preference for crystal orientation (figure not shown), because the ratio of major to minor axis length is close to one when ice crystals are small (e.g., Auer & Veal, 1970; Heymsfield, 1972; Heymsfield & Iaquinta, 2000).

Based on the spatial distribution of *η*_*z*_ (or Figure 12) and the difference in HOIC formation between HV07C and HV11W, the spatial distribution of thin clouds with HOICs can be inferred against the CALIPSO observations as follows. (1) Since *η*_*z*_ < 1 below 10 km and *η*_*z*_ > 1 above 10 km in the tropics and middle latitudes (Figure 12), HV07C and HV11W predict that the thin clouds below 10 km have more HOICs than those above 10 km, which is consistent with the CALIPSO observations. The statistical analysis of the CALIPSO observations reveals that most of the HOICs in thin clouds occur below 10 km altitude (or cloud temperature above ‐30°C), whereas few above 10 km (Noel & Chepfer, 2010; Zhou et al., 2012), which coincides with the vertical distribution of *η*_*z*_ in the tropics and middle latitudes. (2) Since *η*_*z*_ usually decreases with latitude (Figure 12), it is inferred that that the frequency of thin clouds with HOICs increases with latitude, which is consistent with the statistical results of CALIPSO, too (Noel & Chepfer, 2010).

The aircraft observations of precipitating ice crystals (Braham & Spyers‐Duran, 1967) also support the prediction of HV07C and Figure 12. Since *η*_*z*_ < 1 below 10 km in the middle and low latitudes (Figure 12), precipitating ice crystals in the clear sky lose energy efficiently to space and consequently their temperature is much lower than their environmental temperature. As a result, their sublimation rate in dry air is decreased significantly so that they can survive a fall of kilometers through relatively dry air in the upper troposphere once they fall from cirrus clouds (Stephens, 1983).

In the high latitudes, ${\left(\eta_{z}\right)}_{\text{background}}$ < 1 throughout the troposphere, especially during wintertime (Figure 12), indicating that all of the ice crystals in thin clouds undergo radiative cooling there. Hence, the REM model predicts that precipitating thin ice clouds are common in the Arctic, which corresponds to the high frequency of diamond dust observed there (Zeng, 2018b). The REM model also predicts that large ice crystals grow while small ones shrink, which is supported by the observation of Goerke et al. (2017). Goerke et al. (2017) used two‐dimensional reflections of a lidar beam to determine the pattern of individual ice crystals (e.g., ice crystal shape, size, roughness, and orientation) and discovered that small ice crystals sublimate while large ones grow by vapor deposition in an air parcel. Their unique observations agree well with the REM prediction.

## 5 Discussions

The preceding analyses address REM and HOICs in stationary air parcels, such as those near cloud top and the residue left after cloud dissipation. In this section, other factors of HOIC are discussed, beginning with the vertical velocity *w*.

### 5.1 Vertical Velocity

**Figure 13:**
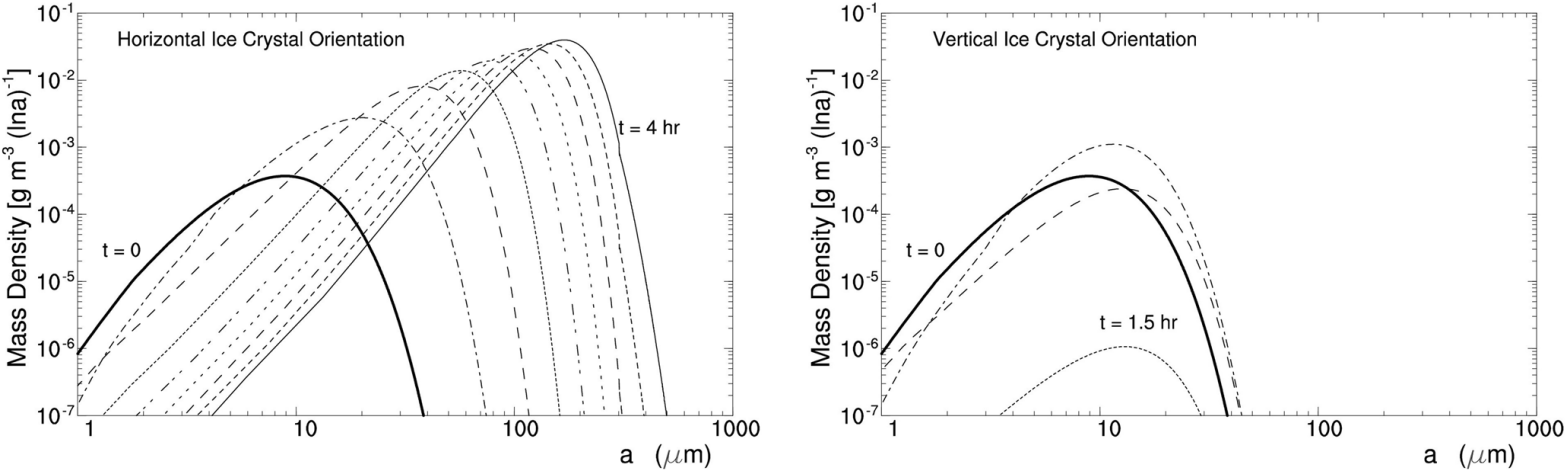
Same as Figure 9 except for HV07CU with a vertical velocity of *w* = 5 mm/s.

Upward motion can accelerate the formation of HOICs via REM, which is shown by an experiment of HV07CU. HV07CU uses the same setup as HV07C except for the vertical velocity *w* = 5 mm/s. Its modeled ice crystal spectrum is displayed in Figure 13. Generally speaking, HV07CU generates large HOICs just as HV07C but yields much more large HOICs than the latter. In other words, many large HOICs form in expense of the water vapor available for deposition due to the upward motion in HV07CU, showing that upward motion can accelerate the formation of precipitating HOICs via REM.

In contrast, the vertically oriented ice crystals in HV07CU are more than those in HV07C near 1 h. If the vertical velocity *w* is increased, the number concentration of vertically oriented ice crystals at 1 h is increased correspondingly, even though the number concentration is still much lower than that of HOICs.

The effect of upward motion on HOICs is understandable. In a stationary air parcel (e.g., HV07C), large ice crystals grow in expense of water vapor, whereas small ones shrink to supply water vapor, maintaining a quasi‐balance in water vapor between the large and small ice crystals. Once an upward motion occurs (e.g., HV07CU), it changes the quasi‐balance by providing additional water vapor for deposition. To be specific, the upward motion increases supersaturation so that large HOICs grow fast while the small ones need no sublimation to supply water vapor.

This modeling result of large HOICs is consistent with the GMI observation: high 166PD over the cloud anvil near mesoscale convective systems (MCSs). Figure 1 displays a MCS near 4°N, where 166PD over its anvil is quite high. A similar case was presented in Zeng et al. (2019) where CloudSat flew right over the anvil of a MCS. Since the cloud anvils near convective cores usually have weak upward motion in the upper troposphere (Zeng et al., 2013), the observation of high 166PD over the anvils near MCSs is consistent with the modeling of large HOICs in HV07CU.

### 5.2 Ice Crystal Shape

**Figure 14:**
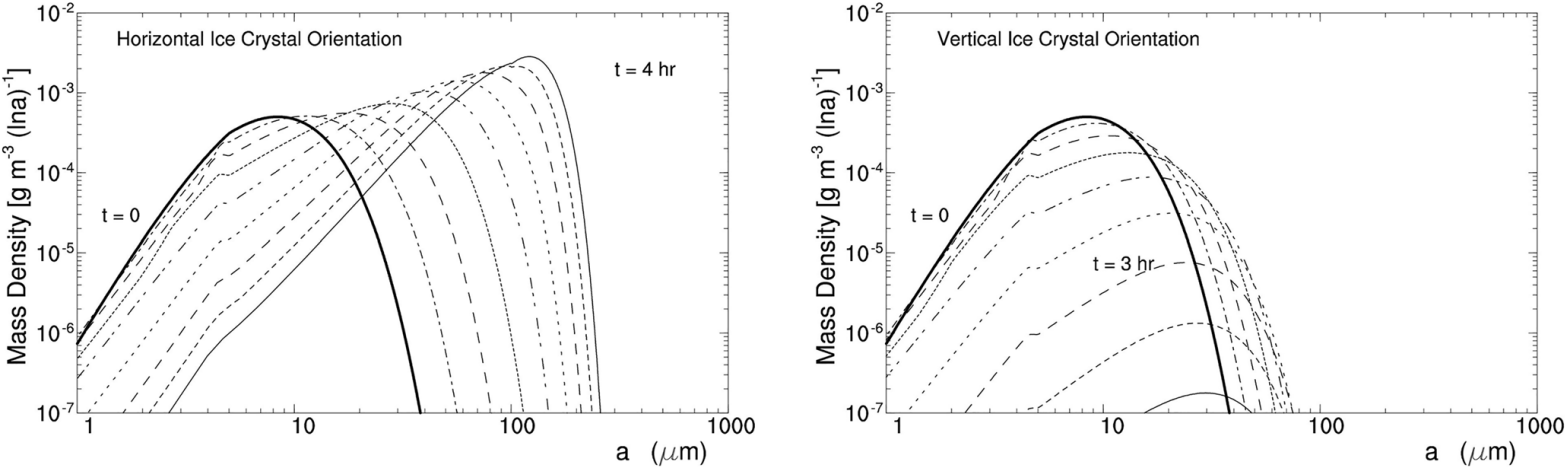
Same as Figure 9 except for HV07CS with column‐like ice crystals.

Ice crystal habit (or shape) varies with temperature and supersaturation (Bailey & Hallett, 2009; Magono & Lee, 1966; Murray et al., 2015). In this subsection, an experiment of HV07CS is carried out to test the sensitivity of REM to ice crystal shape. HV07CS uses the same setup as HV07C except that all ice crystals are column like. Its modeled ice crystal spectrum is displayed in Figure 14. It generates precipitating HOICs just as HV07C. However, its HOIC spectrum is narrower than that in HV07C, indicating that the REM‐induced conversion of water from small to large column‐like HOICs is slower than the counterpart for plate‐like HOICs. In addition, it has more vertically oriented ice crystals than HV07C, showing that the conversion of water from vertically oriented column‐like ice crystals to horizontally oriented ones is slower than the counterpart for plate‐like ice crystals.

In summary, REM for column‐like ice crystals is weaker than that for plate‐like ones. Hence, column‐like ice crystals can bring about lower GMI 166PD than plate‐like ones via REM, given the same IWC. However, how to distinguish the contributions of column‐like and plate‐like ice crystals to GMI 166PD is still a challenge.

### 5.3 Turbulence in the Free Troposphere

Turbulence can disrupt an orientation order that ice crystals would otherwise possess, bringing about no preferred orientation of ice crystals (Klett, 1995). If turbulence is strong, it dominates ice crystal orientation, because it can change ice crystal orientation quickly, with a time scale much shorter than those of other processes (e.g., REM). Such effect of strong turbulence on ice crystal orientation is consistent with the GMI observations. Zeng et al. (2019) statistically analyzed the 166PD over convective and thick clouds. They found that the 166PD over convective clouds is lower than that over thick clouds, which makes sense because the strong turbulence in convective clouds can disrupt any preferred ice crystal orientation.

However, if turbulence is very weak or no turbulence occurs, turbulence has no impact on ice crystal orientation. In fact, the observed turbulence in the stable atmosphere is very weak, although the current observational technologies cannot distinguish the velocity fluctuations from turbulence and gravity waves (Anderson, 2009). Recently the weak turbulence in the stable troposphere was replicated by a turbulence model that incorporates the Monin–Obukhov similarity and the turbulence damping induced by gravity waves (Zeng & Wang, 2020; Zeng et al., 2020). Hence, it is inferred that the free troposphere usually has weak or no turbulence.

If a cirrus cloud had stronger turbulence than its environment for some reason, what would happen? Its ice crystals would be randomly oriented due to its strong turbulence. However, the polarization lidar observations show that HOICs arise near cloud top (Goerke et al., 2017; Neely et al., 2013). In addition, the high frequency of positive 166PD observed by GMI suggests that HOICs are common near cloud top. These observations of HOICs imply that the turbulence in cirrus clouds, at least near cirrus cloud top, is usually weak.

## 6 Conclusions

REM impacts cirrus clouds broadly. In this study, its footprints (e.g., HOICs) are predicted by bin‐model simulations first and then traced in cloud observations. For a practical study, clouds are sorted into several groups according to *η*_*z*_ so that each group has the same REM performance. Then, bin‐model simulations are compared to observations to identify REM in each group. This investigation is carried out with the following points.

- REM is classified into two types: radiative cooling and warming, which are represented by *η*_*z*_ < 1 and >1, respectively. Radiative cooling favors the formation of HOICs but radiative warming does not, as shown by the bin‐model simulations.
- The spatial distribution of *η*_*z*_ (or radiative cooling/warming) is computed with satellite‐retrieved cloud variables (Figure 4). The distribution in thick clouds is quite different from that in thin clouds, suggesting clouds be classified into two kinds: thick and thin.
- Thick clouds have a dipole structure of *η*_*z*_: radiative cooling (or *η*_*z*_ < 1) near cloud top and warming (or *η*_*z*_ > 1) near bottom. Hence, the bin‐model simulations predict that HOICs occur near thick cloud top, which is consistent with the high frequency of positive GMI 166PD over thick clouds (Zeng et al., 2019). The bin‐model simulations also predict more HOICs in midlevel thick clouds than in high‐level ones, which is consistent with the difference in GMI 166PD between midlevel and high‐level clouds (Figure 11; Gong et al., 2018).
- Thin clouds usually undergo radiative cooling below 10 km and warming above 10 km altitude. Hence, the bin‐model simulations predict more HOICs below 10 km than above 10 km, which is consistent with the CALIPSO observations of HOICs (Noel & Chepfer, 2010; Zhou et al., 2012).
- REM depends on not only *η*_*z*_ and cloud height but also vertical velocity and ice crystal shape. The bin‐model simulation shows that upward motion can accelerate the HOIC formation in thick clouds, which is consistent with the high 166PD over the cloud anvils near MCSs.

The general agreement between the bin‐model simulations and cloud observations suggests that REM does exist in many cirrus clouds. The common high GMI 166PD indicates that REM impacts cirrus clouds broadly. In the future, REM with its factors (e.g., *η*_*z*_, *T*, and vertical velocity) will be incorporated into a cloud/radiation parameterization to better simulate clouds in weather and climate models.

## Data Availability

The coincidence satellite data are available from https://pmm.nasa.gov/data-access/downloads/gpm, the satellite‐retrieved cloud data from http://www.cloudsat.cira.colostate.edu/, and the present model output from https://osf.io/4tsn3/.

## Appendix A Ice Crystal Temperature Versus Ice Crystal Size and Orientation

### A.1 Two Radiative Ratios

#### A.1.1

To derive the analytic expressions of ice crystal temperature and growth rate, two radiative ratios of *η*_z_ and *η* are introduced to describe radiative cooling/warming of ice crystals in REM (Zeng, 2008, 2018b). The first ratio, *η*_z_, is introduced to describe the inward radiative flux through parcel boundary normalized by a scale of energy flux *σT* ^4^ (or the blackbody irradiance at temperature *T*). It is simplified to Equation 1 if a two‐stream radiation package is used to compute the upward and downward radiative fluxes. The second ratio, *η*, is introduced to represent the ratio of infrared flux incident on ice crystal surface to the radiative flux emitted by the same ice crystal at the environmental air temperature.

The two ratios are quite different, addressing air parcel and ice crystal, respectively. Since *η* changes from one ice crystal to another, it is used to represent the radiative cooling/warming of an ice crystal in the ice crystal growth equation. In contrast, *η*_z_ is independent of ice crystals in an air parcel and thus can work as a variable in an air parcel model or three‐dimensional climate model.

However, the two ratios are connected by

(A1) $$\left(\eta -1\right)=\alpha \left(\eta_{z}-1\right)$$

where the coefficient *α* depends on ice crystal size, shape, and orientation, and 0 ≤ *α* ≤ 1 (see Zeng, 2018b, for the expression of *α*). For a vertically oriented ice crystal with extremely long axis, *α* = 0; for a spherical ice crystal, *α* = 1/2; and for a HOIC with extremely long axis, *α* = 1. Generally speaking, an ice crystal with horizontal orientation possesses larger *α* than itself with vertical orientation.

The sign of *η* ‐ 1 indicates the radiative cooling/warming of an ice crystal (see Equation A2) and *η* ‐ 1 shares the same sign as *η*_z_ ‐ 1 (see Equation A1). Hence, *η*_z_ is used to indicate the radiative cooling/warming of all ice crystals in an air parcel. To be specific, when *η*_z_ < 1, *η* < 1 so that all ice crystals in an air parcel undergo radiative cooling; otherwise, all ice crystals in an air parcel undergo radiative warming. In the present parcel model, *η*_z_ is used as an index to measure the radiative cooling/warming of ice crystals while the real cooling/warming rate of each ice crystals is calculated with *α*(*η*_z_ ‐ 1), where *α* depends on ice crystal shape, size, and orientation.

#### A.1.2 Radiation‐Induced Difference in Temperature Between Ice Crystal and Its Environmental Air

The radiation‐induced difference in temperature between ice crystal and its environment increases almost linearly with ice crystal size, which is illustrated with a simple model. Consider a spherical ice crystal with radius *r*. It emits and absorbs IR, bringing about an imbalance between inward and outward radiative fluxes. Its net radiative flux from the ice crystal to its environment:

(A2) $$Q_{r}\;\propto \;4\pi r^{2}\left(1-\eta \right).$$

Let *T* and *T*_*s*_ denote the environmental air temperature and ice crystal surface temperature, respectively. Thus, the conductive energy flux from the environment to the ice crystal (Mason, 1971):

(A3) $$Q_{c}\;\propto \;4\pi r\left(T-T_{s}\right).$$

Suppose that the ice crystal stays at a steady state and thus maintains an energy balance between the radiative and conductive heat transfers (after ignoring *Q*_*v*_ the heat exchange between the ice crystal and its environment via water vapor diffusion). Thus, *Q*_*r*_ = *Q*_*c*_. Substituting Equation A2 and Equation A3 into *Q*_*r*_ = *Q*_*c*_ yields the temperature difference between ice crystal and its environmental air:

(A4) $$T_{s}-T\propto r\left(\eta -1\right).$$

Obviously, the positive correlation between *T*_*s*_−*T* and *r* in Equation A4 originates in the different sensitivities of *Q*_*r*_ and *Q*_*c*_ to *r*: *Q*_*r*_ is proportional to the ice crystal surface area 4*πr* ^2^, whereas *Q*_*c*_ is proportional to the ice crystal size *r*. Furthermore, substituting Equation A1 into Equation A4 yields a relationship of *T*_*s*_ to ice crystal shape and orientation, showing *T*_*s*_ as a function of *α* or ice crystal orientation.

After incorporating *Q*_*v*_ the heat exchange between the ice crystal and its environment via water vapor diffusion, the energy balance becomes *Q*_*r*_ = *Q*_*c*_ + *Q*_*v*_. Thereafter, an analytical expression of *T*_*s*_−*T* was obtained for spherical and nonspherical ice crystals (Zeng, 2008). Although the analytical expression of *T*_*s*_−*T* looks a little complicated, it still retains the essence of Equation A4: the positive correlation between *T*_*s*_−*T* and ice crystal size.
